# Postoperative pain behaviours in rabbits following orthopaedic surgery and effect of observer presence

**DOI:** 10.1371/journal.pone.0240605

**Published:** 2020-10-22

**Authors:** Renata Haddad Pinho, Matthew C. Leach, Bruno Watanabe Minto, Fabiana Del Lama Rocha, Stelio Pacca Loureiro Luna

**Affiliations:** 1 Department of Anesthesiology, Botucatu Medical School, São Paulo State University (Unesp), Botucatu, São Paulo, Brazil; 2 School of Natural and Environmental Science, Newcastle University, Newcastle upon Tyne, United Kingdom; 3 Department of Veterinary Clinics and Surgery, School of Agricultural and Veterinary Sciences, São Paulo State University (Unesp), Jaboticabal, São Paulo, Brazil; 4 Department of Veterinary Surgery and Animal Reproduction, School of Veterinary Medicine and Animal Science, São Paulo State University (Unesp), Botucatu, São Paulo, Brazil; University of Bari, ITALY

## Abstract

Rabbits are widely used in studies focusing on pain. However, pain is undertreated in this species and one possible factor to explain this is the lack of evaluation methods. The objective of this study was to identify behaviours related to orthopaedic pain in rabbits and to evaluate the influence of the presence of an observer on these behaviours. Twenty-eight rabbits undergoing orthopaedic surgery and filmed 24 hours before surgery, and 1 hour (before rescue analgesia), 4 hours (3 hours after rescue analgesia), and 24 hours post-recovery were observed in the presence and absence of an observer. The frequency and/or duration of behaviours were compared over time and between the presence and absence of the observer using the Friedman and Wilcoxon tests respectively. Data are expressed as median and interquartile range and a significant difference was considered when p<0.05. At 1 hour post-recovery, the rabbits showed reduced activity, hopping, change posture, position in the cage, explore, and open eyes in both the presence and absence of the observer. In the absence of the observer, quadrupedal posture, interact with pinecone, and eat carrot also decreased, while wince behaviour increased. In the presence of the observer, before surgery, the rabbits were less active (Presence-280; 162–300, Absence-300; 300–300) and presented a lower duration of explore (Presence-3; 0–32, Absence-40; 4–63). Post-recovery the rabbits flinched less (Presence-0; 0–0, Absence-0; 0–1) and suspended the affected limb less (Presence-0; 0–0, Absence-0; 0–65). After rescue analgesia the rabbits put weight on and raised the affected limb less (Presence-0; 0–0, Absence-0; 0–2) and licked the affected area less (Presence-0; 0–0, Absence-0; 0–2). These findings demonstrate that the presence of the observer inhibited pain-free behaviours in the rabbits, leading to a false impression of pain, and after the surgery the rabbits masked some pain signs related to the affected area.

## Introduction

Pain presents sensory, emotional, cognitive, and social components [1]. Rabbits *(Oryctolagus cuniculus)* are widely used worldwide as experimental models. In the UK alone, more than 11,000 experimental procedures involving rabbits were performed in the year 2018 [2].

Although rabbits and other laboratory animals are fundamental for translational medicine, paradoxically, pain in these species is still neglected [3]. Postoperative administration of analgesics to rodents and lagomorphs following experimental procedures reaches only 10.4% of the studies [4], and one possible reason for this is the difficulty in identifying and quantifying pain [5, 6], due to the lack of a simple and accurate method to evaluate acute pain in laboratory animals [7].

Methods routinely used to evaluate pain in rabbits include those based on evoked responses to a mechanical, thermal, or electrical nociceptive stimulus [8], however, these are not normally applicable to clinically evaluate postoperative pain, since they do not reflect spontaneous pain and are hard to implement. Although other methods, such as a reduction in body weight, and feed and water intake can also reflect pain states [7], these are retrospective indicators demonstrating that the animal has felt pain and, as they do not represent the animal’s current state, and so do not serve as a guide to the necessity to administer analgesics [9].

Thus, the most applicable methods for real-time clinical pain measurement are based on evaluation of behaviour. The Rabbit Grimace Scale (RbtGS) [10] is a facial expression based scale developed and evaluated in rabbits submitted to ear tattooing. A recent scale was developed to assess pain in pet rabbits by merging this facial scale with physiological and behavioural parameters [11].

An ethogram offers an appropriate starting point to identify pain behaviours [9, 10, 12–14], as it is a record of terms and descriptions of behaviour of an individual during a given period and situation [14]. In order to develop an ethogram to identify pain behaviours, one must compare behaviours present in situations that incur pain with behaviours present in a pain-free state, usually before surgery [9, 13]. Ethograms have only been developed in rabbits suffering soft tissue surgery [9, 12, 13, 15], and to our knowledge, only activity levels, not behaviour, have been evaluated in rabbits undergoing orthopaedic surgery [8]. Orthopaedic pain is expected to induce more intense pain [16, 17] and to be associated with specific behaviours (e.g. lameness) [18] in addition to those already observed [9, 12, 13, 15]. The impact of orthopaedic surgery is of particular concern and requires investigation as rabbits are frequently submitted to this procedure both clinically as pets [17] and in research [16].

Changes in general behaviour that have been associated with pain in rabbits and can include: reductions in activity [8, 9], moving around [9, 13, 15], hopping [9, 13], exploration [9, 13], and interaction with objects and other animals [9] as well as increased lying posture [9, 12]. Changes in behaviours considered to be more specific to abdominal pain have also been observed, such as, skin twitches, writhing and pressing the abdomen against the floor [9].

Although behaviour is a well-established method for evaluating acute pain in animals and widely used in many species [6, 19–25], the interaction or presence of a human observer may interfere with evaluations [26], and rabbits, like ruminants and rodents, tend to mask their pain behaviours [3, 5, 6, 19, 27]. To date, pain behaviour has often been described in rabbits [9, 12, 13] and guinea pigs [19] in the absence of a human observer, however, no studies have compared the effect of observer presence and absence on the exhibition pain behaviour in rabbits. In rats, scores based on facial expressions decreased in the presence of a male observer [28], while the latency to exhibit behavioural expression of pain increased in the presence of a cat [29]. Some authors have inferred that rabbits avoid demonstrating pain behaviour in the presence of an observer [3, 9], however this supposition has not been scientifically evaluated.

Consequently, it is crucial to identify the degree to which the presence of an observer influences the exhibition of rabbit behaviours associated with pain and whether it is possible to assess pain in-person or if a remote assessment is necessary to effectively identify pain behaviours in this species.

The current study aimed to determine the frequency and duration of acute pain-related behaviours in rabbits undergoing orthopaedic surgery and to compare them when a human observer was present or absent, based on the hypothesis that pain modifies the behavioural state of normality of rabbits and the presence of a human observer reduces the expression of pain-free and painful behaviours.

## Materials and methods

The study was approved by the Ethical Committee for the Use of Animals in Research, of the School of Veterinary Medicine and Animal Science, of São Paulo State University (Unesp) and School of Agricultural and Veterinary Sciences, São Paulo State University (Unesp), under protocol numbers 0156/2018 and 019155/17 respectively. The study follows the Brazilian Federal legislation of CONCEA (National Council for the Control of Animal Experimentation) and covered two of the principles of the 3 R’s (reduction and refinement), referring to good practice in animal experimentation [30]. This is an opportunistic study (reduction) conducted in conjunction with another unrelated study, in which the animals underwent partial radius ostectomy, to identify pain behaviours in rabbits (refinement).

### Animals

Twenty-eight New Zealand rabbits (*Oryctolagus cuniculus*) from the Central Vivarium of UNESP (São Paulo State University, Botucatu) were used, including 11 females and 17 males, 159±5 days old, and mean weight of 3.7±0.38kg.

As inclusion criteria, healthy animals were selected after laboratory (haemogram) and physical exams, including inspection, cardiac and respiratory auscultation, and rectal temperature measurement. Animals would be excluded from the study if they presented complications during surgery, such as bleeding, hypotension (systolic arterial blood pressure < 80 mmHg) or bradycardia (heart rate < 130 bpm) not resolved by reducing the concentration of the volatile anaesthetic or by fluid administration, arrhythmias, or, after the surgical procedure, complications such as intestinal motility disorders.

The animals were allocated to an experimental rabbit shelter adapted to house experimental animals, with natural ventilation controlled by curtains. Each rabbit was kept in an individual stainless steel cage, 60cm³ in size, with a grid floor litter tray which was cleaned twice a day. The study was conducted between August and September, with a local natural photoperiod of approximately 12 hours of light per day and a mean temperature of 21°C. The adaptation of the housing occurred approximately 40 days before surgery. Four days before the video recording, the animals were housed in specific cages within the same shelter to acclimate.

Water and dry feed (Fri-Coelho, FRI-RIBE, Trouw Nutrition, São Paulo, SP, Brazil) were supplied *ad libitum* in open dish drinkers and feeders, as well as carrot pieces daily and Tifton hay (*Cynodon spp*.) was provided twice a week. To supply the rabbits’ needs for interaction and chewing, eucalyptus pinecones were also provided.

### Anaesthesia and surgery

Two days before surgery, the animals were anaesthetized through isoflurane inhalation (Isoforine 100%. Cristália Pharmaceutical Products LTDA, São Paulo, SP, Brazil) at a concentration of 4–5 Vol% diluted in oxygen 4L/min administered in an induction chamber, followed by a face mask. The superficial anaesthetic plane was maintained, confirmed by the presence of foot reflexes and ear pinching for a period of three to five minutes, to allow shaving the surgical region (right thoracic limb), the ears (for venous access), and the distal region of the left thoracic limb (for monitoring arterial blood pressure with a Doppler ultrasound).

On the day of surgery, the non-fasting rabbits were treated with 5 mg/kg pethidine (Dolosal 50mg/mL, Cristália Pharmaceuticals LTDA, Sao Paulo, SP, Brazil) via the lumbar epaxial intramuscular (IM) route. Next, a 24G catheter was inserted into the marginal ear vein and fluid therapy was instigated with lactated Ringer’s solution (RL) at a rate of 3mL/kg/h administered by a syringe infusion pump. Oxygen therapy was instituted at 2L/min via face mask for three minutes and anaesthetic induction with 3 to 5 Vol% isoflurane diluted in 2L/min oxygen. Following the absence of interdigital and ear pinching reflexes, 0.1 mL of lidocaine (Xylestesin 2% without vasoconstrictor, Cristália Pharmaceuticals LTDA, São Paulo, SP, Brazil) was instilled into the arytenoids region, maintaining inhalation of isoflurane for a further minute. Capnography-guided orotracheal intubation (Capnostat 5, Digicare Animal Health, Rio de Janeiro, RJ, Brazil) was performed with an endotracheal tube of internal diameter 2.5–3.5mm, compatible with the size of the animal. If, after three attempts, intubation was not possible, anaesthesia was maintained via the face mask. The inspired isoflurane concentration was adjusted to maintain the surgical anaesthetic plane and 2 μg/kg of fentanyl (Fentanest 0.0785 mg/mL, Cristália Pharmaceuticals LTDA, Sao Paulo, SP, Brazil) was administered intravenously (IV) immediately prior to the start of surgery.

Anaesthetic monitoring was recorded on an appropriate chart every five minutes, consisting of heart rate, peripheral haemoglobin oxygen saturation, respiratory rate, expired CO_2_ fraction (EtCO_2_) (LifeWindow LW9x, Digicare Animal Health, Rio de Janeiro, RJ, Brazil), rectal temperature using a digital thermometer, and non-invasive systolic arterial blood pressure with a Doppler ultrasound (Vascular Doppler model 811-B. Parks Medical Inc, OR, USA), in addition to identifying the beginning and end of surgery and possible anaesthetic complications.

Prior to surgery, limb antisepsis was performed with 2% chlorhexidine followed by 0.5% alcohol chlorhexidine. An incision of approximately 2.5 cm was made in the skin of the craniomedial region of the radius, followed by blunt dissection of subcutaneous tissue and musculature, which allowed exposure of the distal radius diaphysis. Partial ostectomy was performed of 1 cm extension in the radial diaphysis, 1.5 cm distant from the radiocarpal joint, using an oscillatory saw and a spectrometer for measurements. The segment of the radius was removed together with the periosteum and the wound was sutured. The surgeries were performed between 8am and 4pm and each surgical procedure took between 10 and 30 minutes. Anaesthetic recovery was defined as when the rabbits, positioned in sternal recumbency, were able to support the head.

One hour after anaesthetic recovery, all animals received rescue analgesia with 2 mg/kg morphine (Dimorf 1%, Cristália Pharmaceuticals LTDA, Sao Paulo, SP, Brazil) and 1 mg/kg meloxicam (Maxicam 2%, Ourofino, Sao Paulo, SP, Brazil) IM. Eight hours after anaesthesia recovery the animals were evaluated according to the researcher’s clinical assessment and if necessary, an extra dose of 1 mg/kg of morphine was administered, IM. From 24 hours after anaesthetic recovery, the animals received 5 mg/kg tramadol hydrochloride (Tramadol hydrochloride, Teuto, Goiás, Brazil) IM every 8 hours for three days.

### Video recordings

**Fig 1:**
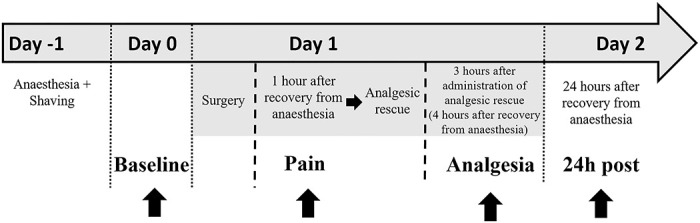
Timeline of procedures and video recordings.

The twenty-eight animals were filmed at I) 24 hours before surgery (Baseline), II) 1 hour after recovery from anaesthesia (Pain), III) 3 hours after rescue analgesia, i.e. 4 hours after recovery from anaesthesia (Analgesia), and IV) 24 hours after recovery from anaesthesia (24h post) (Fig 1).

The same female researcher (RHP), who was familiar to the rabbits, made all of the recordings. After positioning the camera, a piece of carrot, approximately 2 x 2.5 cm, was offered, the pinecone already present was removed and replaced with a new one, and then the observer left the room. The observer waited in silence in another room about three meters away from the room housing the rabbits. After five minutes (to allow the rabbit to acclimate to the researcher’s absence), the camera was triggered using a mobile app (GoPro App. GoPro Inc., California, USA) and filming continued for five minutes (absence of observer). At the end of this five minutes of filming, the researcher returned to the filming room, sat in a chair in front of the cage behind the camera and filming continued for another five minutes (presence of the observer).

### Ethogram

The ethogram was developed and adapted according to the pain behaviours described after ovariohysterectomy (OVH) [9] as a reference. The video sequences recorded were first analyzed to identify whether the same pain behaviours as observed after OVH were present in this study. This initial analysis also allowed the identification of new pain behaviours that were not previously reported after OVH [9], these included: ‘scissor’ ears, press limbs, scratch ears, punch, eat a carrot, interact with the pinecone, ingest cecotropes, tremble, suspend limb, put weight on and raise the affected limb, and try to get up. Semantical adaptations were also made to the original ethogram [9]. Some descriptions were altered according to the state and are described in Table 1.

**Table 1:** Modifications from the original ethogram developed for rabbits submitted to OVH [9].

Behaviour	Original description	Current description
Normal posture	Sitting relaxed with hind limbs tucked under the rump and fore limbs underneath	Flexes pelvic limbs under the hip, thoracic limbs under the body; abdomen and chest not supported on cage floor
React	Originally named “alert”: Immediate reaction of individual to being disturbed; animal momentarily looks around	Moves head and/or body sharply in response to environmental stimulus (e.g., sound stimulus)
Wince	Rocking motion accompanied by eye closing and swallowing action	Shrinks back and closes eyes
Twitch	Rapid movement of fur on back	Presents spasms in the skin of the back

The following behavioural categories were considered: activity, position in cage, posture, position of the ears in relation to the body, eye opening, typical behaviours, interaction with motivational items, physiological behaviours (eat, drink, and ingest cecotropes), self-cleaning, and pain-related behaviours (see Table 2).

**Table 2:** Ethogram of rabbits undergoing orthopaedic surgery.

Behaviour	Description	F	D
*1*. *Activity*			
Active	Moves and/or moves around		**✓**
Inactive	Remains stationary		**✓**
*2*. *Position in the cage*			
Front	Front of the cage		**✓**
Back	Back of the cage		**✓**
Change position	Moves around the cage: front to back or back to front	**✓**	
*3*. *Posture*			
Normal	Flexes pelvic limbs under the hip, thoracic limbs under the body; abdomen and chest not supported on cage floor		**✓**
Seated	Sits with vertically extended thoracic limbs		**✓**
Lying down	Lies with the abdomen and chest in contact with the cage floor and horizontally extended thoracic limbs		**✓**
Lying on one side	Lies on one side of the body with horizontally extended thoracic limbs		**✓**
Quadrupedal	In the quadrupedal position with the four limbs extended vertically; abdomen not in contact with the cage floor		**✓**
Bipedal	Supported on both pelvic limbs; thoracic limbs not touching the floor	**✓**	**✓**
Change posture	Alternates between different postures	**✓**	
*4*. *Position of the ears in relation to the body*			
Completely lowered	Parallel to the vertebral column		**✓**
Semi-lowered	Semi-lowered (positioned between fully lowered and erect)		**✓**
Erect	Erect, perpendicular to the spine		**✓**
‘Scissor’ ears	Each ear moves to different positions		**✓**
*5*. *Eye opening*			
Open	Fully open		**✓**
Semi-closed	Semi-closed		**✓**
Closed	Closed		**✓**
Not visible	Not possible to visualize the eyes		**✓**
*6*. *Typical rabbit behaviours*			
Hop	Hops to move around with both pelvic limbs at the same time	**✓**	
Rotating jump	Jumps performing a 180° or 360° rotation with both pelvic limbs at the same time	**✓**	
React	Moves head and/or body sharply in response to environmental stimulus (e.g., sound stimulus)	**✓**	
Shake body	Shakes the whole body	**✓**	
Shake the head	Shakes only the head	**✓**	
Dig	Digs the floor	**✓**	**✓**
Explore	Sniffs the cage floor and/or walls and/or bars curiously	**✓**	**✓**
Gnaw	Gnaws the floor or bars of the cage	**✓**	**✓**
Press limbs	Presses limbs strongly against the cage floor	**✓**	
Stretch	Stretches the body	**✓**	
Scratch ear	Scratches the ears with the limbs	**✓**	**✓**
Punch	Extends the thoracic limbs horizontally, quickly alternating between right and left	**✓**	
*7*. *Interaction with motivational items*			
Interact with pinecone	Interacts with the pinecone (chews, gnaws, pushes with the thoracic limbs)	**✓**	**✓**
Eat carrot	Eats the carrot	**✓**	**✓**
*8*. *Physiological behaviours*			
Drink	Drinks water from the water trough	**✓**	**✓**
Eat feed	Eats feed from the feeder	**✓**	**✓**
Ingest cecotropes	Ingests anal cecotropes	**✓**	**✓**
*9*. *Self-cleaning*: *by licking; possibly with assistance from the thoracic limbs*			
Head	Head and ears	**✓**	**✓**
Body	Body, including abdomen and limbs (except affected limb)	**✓**	**✓**
*10*. *Pain-related behaviours*			
Lick affected area	Licks affected region	**✓**	**✓**
Twitch*	Presents spasms in the skin of the back	**✓**	
Flinch*	Moves body quickly dorsally and for no apparent reason	**✓**	
Wince*	Shrinks back and closes eyes	**✓**	
Stagger*	Partially loses balance	**✓**	
Fall*	Totally loses balance; falls to the floor	**✓**	
Tremble	Presents tremors observed in head and ears	**✓**	**✓**
Suspend limb	Keeps the affected limb suspended	**✓**	**✓**
Put weight on and raise the affected limb	Raises and puts weight on the affected limb repeatedly	**✓**	
Try to get up	Tries to get up, but remains in a normal posture or lying down	**✓**	
Writhe*	Contracts the muscles of the abdomen	**✓**	

F: frequency; D: duration.

* indicates pain behaviours taken from [9].

The video examples of each behaviour are available in the S1 Table.

Once the behaviours had been identified in the first analysis of the videos, the footage was evaluated again to calculate the frequency of ‘event behaviours’ (number of occurrences started) and the duration (in seconds) of all behaviours (i.e. ‘state’ behaviours). The duration was recorded in seconds for behavioural states and only frequency was reported for event behaviours. Duration and frequency were assessed for some behaviours, such as self-cleaning and interaction with pinecones and carrots (Table 2).

### Statistical analysis

Taking into account a test power of 90% with an alpha of 5% (http://biomath.info/power), 21 rabbits were necessary to observe differences in duration of activity at baseline, when the observer was present compared to when the observer was absent, and 11 rabbits were necessary to compare duration of activity between the baseline and 1h post-recovery time points in the presence of the observer.

Analysis were performed using Graph Pad Prism 5.0 software considering an α of 5%. According to the Shapiro Wilk test and histogram distribution analysis, data were defined as non-normal. To compare the time-points (Baseline, Pain, Analgesia, and 24h post) Friedman’s test was used followed by Dunn’s multiple comparison post-hoc test. To compare the behaviours between the presence and absence of the observer, the Wilcoxon test for paired samples was used. Differences were considered statistically significant when p<0.05.

## Results

None of the rabbits were excluded from the study. It was not possible to perform endotracheal intubation in three animals and, therefore, they were kept under inhalation anaesthesia via a face mask. EtCO_2_ was greater than 45mmHg in all rabbits at some time-point during anaesthesia, which was resolved by manual assisted ventilation or by reducing isoflurane concentration. Nine rabbits had transient hypotension (systolic arterial blood pressure < 80 mmHg) which was successfully treated by reducing the concentration of isoflurane in eight rabbits or by administering fluids (10 ml/kg of Lactated Ringer in 10 minutes) in one rabbit.

Rabbits recovered 3±2.8 (1–13) minutes after the end of anaesthesia. There were no cases of postoperative hypothermia or other complications. Three rabbits received an additional administration of 1mg/kg of morphine eight hours after recovery from anaesthesia.

In total, 50 behaviours were identified. Thirty nine behaviours had been previously described in rabbits undergoing OVH [9] and an additional 11 new behaviours were identified in the current study (‘scissor’ ears, press limbs, scratch ears, punch, eat a carrot, interact with the pinecone, ingest cecotropes, tremble, suspend limb, put weight on and raise the affected limb, and try to get up) (Table 2).

The frequency and/or duration of 19 and 13 of these 50 behaviours respectively changed over time and from the absence to presence of the observer. The medians and interquartile range of the duration and frequency of occurrence of each behaviour that altered over time or was affected by the presence of the observer are described in Table 3.

**Table 3:** Median and interquartile range (Q1 –Q3) of behaviour of 28 rabbits that altered over time or between the presence and absence of observer.

Behaviour	F/D	Observer	Baseline	Pain	Analgesia	24h post
*Activity*						
Active	D	Pr	280 (162–300)^a^	0 (0–78)^b^	0 (0–16)^b^	300 (92–300)^a^
		Ab	300 (300–300)^a^^†^	11 (0–300)^b^^†^	0 (0–136)^b^^†^	300 (300–300)^a^^†^
Inactive	D	Pr	20 (0–138)^b^^†^	300 (222–300)^a^^†^	300 (284–300)^a^^†^	0 (0–208)^b^^†^
		Ab	0 (0–0)^b^	289 (0–300)^a^	300 (164–300)^a^	0 (0–0)^b^
*Position in cage*						
Front	D	Pr	204 (91–289)^a^	0 (0–300)^ab^	0 (0–139)^b^	229 (62–291)^a^
		Ab	161 (72–270)	0 (0–295)	0 (0–220)	196 (110–265)
Back	D	Pr	96 (12–209)^b^	300 (0–300)^ab^	300 (161–300)^a^	71 (9–238)^b^
		Ab	139 (31–229)	300 (5–300)	300 (81–300)	105 (35–190)
Change position	F	Pr	2 (1–5)^a^	0 (0–0)^c^	0 (0–0)^bc^	1 (0–2)^ab^
		Ab	4 (0–7)^a^	0 (0–0)^b^	0 (0–0)^b^	1 (0.3–5)^a^
*Posture*						
Normal	D	Pr	91 (8–199)	63 (0–300)	15 (0–237)	50 (2–143)
		Ab	142 (71–249)^a^	117 (0–300)^ab^	0 (0–229)^b^	157 (24–221)^ab^^†^
Seated	D	Pr	128 (10–220)^a^	0 (0–212)^ab^	0 (0–0)^b^	131 (44–236)^a^
		Ab	57 (3–162)	0 (0–216)	0 (0–170) ^†^	111 (44–253)
Lying down	D	Pr	0 (0–0)^b^	0 (0–262)^ab^	241 (0–300)^a^	0 (0–64)^b^^†^
		Ab	0 (0–0)^b^	0 (0–109)^ab^	62 (0–300)^a^^†^	0 (0–0)^b^
Quadrupedal	D	Pr	0 (0–38)	0 (0–0)	0 (0–0)	0 (0–0)
		Ab	0 (0–91)^a^	0 (0–0)^b^	0 (0–0)^ab^	0 (0–17)^ab^
Change posture	F	Pr	2 (1–6)^a^	0 (0–1)^b^	0 (0–1)^b^	3 (1–8)^a^
		Ab	4 (1–9)^a^	0 (0–1)^b^	0 (0–2)^b^	3 (1–7)^a^
*Eye opening*						
Open	D	Pr	293 (237–300)^a^	57 (7–194)^c^	60 (12–282)^bc^	255 (57–300)^ab^
		Ab	286 (206–300)^ab^	10 (0–59)^c^	3 (0–137)^bc^	265 (192–300)^a^
Semi-closed	D	Pr	0 (0–0)^b^	94 (2–208)^a^	69 (0–208)^a^	0 (0–87)^ab^
		Ab	0 (0–0)^b^	215 (0–294)^a^	109 (13–285)^a^	0 (0–0)^b^
*Typical rabbit behaviours*						
Hop	F	Pr	2 (1–6)^a^	0 (0–0)^b^	0 (0–0)^b^	1 (0–4)^a^
		Ab	4 (1–14)^a^	0 (0–0)^b^	0 (0–0)^b^	3 (0–6)^a^
React	F	Pr	0 (0–1)	0 (0–0)	0 (0–0)	0 (0–1)^†^
		Ab	0 (0–0)	0 (0–0)	0 (0–0)	0 (0–0)
Shake body	F	Pr	0 (0–0)	0 (0–0)	0 (0–0)	0 (0–0)
		Ab	0 (0–1)	0 (0–0)	0 (0–0)	0 (0–1) ^†^
Explore	F	Pr	1 (0–2)^a^	0 (0–0)^b^	0 (0–0)^ab^	0 (0–1)^ab^
		Ab	3 (1–4)^a^^†^	0 (0–0)^b^	0 (0–0)^b^	0 (0–2)^ab^
	D	Pr	3 (0–32)^a^	0 (0–0)^b^	0 (0–0)^ab^	0 (0–17)^ab^
		Ab	40 (4–63)^a^^†^	0 (0–0)^b^	0 (0–0)^b^	0 (0–20)^b^
*Interaction with motivational items*						
Interact with the pinecone*	F	Ab	2 (0–4)^a^	0 (0–0)^b^	0 (0–0)^b^	1 (0–2)^a^
	D	Ab	15 (0–64)^a^	0 (0–0)^b^	0 (0–0)^b^	8 (0–63)^a^
Eat the carrot*	F	Ab	1 (0–1)^ab^	0 (0–0)^c^	0 (0–0)^ac^	1 (0–1)^b^
	D	Ab	21 (0–97)^ab^	0 (0–0)^bc^	0 (0–0)^c^	97 (9–201)^a^
*Self-cleaning*						
Body	F	Pr	0 (0–2)	0 (0–0)	0 (0–0)	1 (0–4)
		Ab	1 (0–3)	0 (0–0)	0 (0–0)	0 (0–2)
	D	Pr	0 (0–20)^ab^	0 (0–0)^b^	0 (0–0)^ab^	3 (0–37)^a^
		Ab	1 (0–12)	0 (0–0)	0 (0–0)	0 (0–17)
*Pain-related behaviours*						
Lick affected area	F	Pr	0 (0–1)	0 (0–1)	0 (0–0)	1 (0–2)
		Ab	0 (0–1)	0 (0–3)	0 (0–1)	0 (0–1)
	D	Pr	0 (0–3)	0 (0–2)	0 (0–0)	1 (0–12)
		Ab	0 (0–2)	0 (0–111)	0 (0–2)^†^	0 (0–7)
Flinch	F	Pr	0 (0–0)	0 (0–0)	0 (0–0)	0 (0–0)
		Ab	0 (0–0)	0 (0–1) ^†^	0 (0–0)	0 (0–0)
Wince	F	Pr	0 (0–0)	1 (0–1)	0 (0–0)	0 (0–1) ^†^
		Ab	0 (0–0)^b^	1 (0–1)^a^	0 (0–1)^ab^	0 (0–0)^ab^
Suspend limb	F	Pr	0 (0–0)	0 (0–0)	0 (0–0)	0 (0–0)
		Ab	0 (0–0)	0 (0–1)	0 (0–0)	0 (0–1) ^†^
	D	Pr	0 (0–0)	0 (0–0)	0 (0–0)	0 (0–0)
		Ab	0 (0–0)	0 (0–65) ^†^	0 (0–0)	0 (0–1)
Put weight on and raise the affected limb	F	Pr	0 (0–1)^b^	0 (0–0)^b^	0 (0–0)^b^	4 (1–11)^a^
		Ab	0 (0–0)^b^	0 (0–1)^ab^	0 (0–2)^ab^^†^	2 (0–7)^a^

Frequency (F) in number of occurrences and duration (D) in seconds in each of the 300 second (five minute) observations of behaviours in the twenty-eight rabbits in the moments before surgery (Baseline); 1 hour after recovery from anaesthesia (Pain); 4 hours after recovery from anaesthesia and 3 hours after rescue analgesia (Analgesia); and 24 hours after recovery from anaesthesia (24h post). Different letters indicate statistical difference between the time-points according to the Friedman test (p<0.05), being a>b>c. The outline highlighted in the table indicates difference between the observer’s presence (Pr) and absence (Ab);

^†^ indicates significant difference in duration or frequency between Pr and Ab by the Wilcoxon test (p<0.05).

*Data on the behaviours of “eat the carrot” and “interact with the pinecone” in the presence of the observer were not included, because these motivational items were not replaced when the observer entered and remained in the room; therefore the carrot could have already been eaten and the pinecone would not be a novelty factor.

### Pain behaviours related to surgery

Both in the presence (Pr) and in the absence (Ab) of the observer, one hour post-recovery (Pain), the rabbits became less active (Pr and Ab—p < 0.0001), and therefore more inactive (Pr and Ab—p < 0.0001), changed their cage position (Pr and Ab—p < 0.0001), and posture less (Pr and Ab—p < 0.0001), hopped (Pr and Ab—p < 0.0001), and explored (Pr and Ab—p < 0.0001) less, kept their eyes open for a shorter time (Pr—p < 0.001 and Ab—p < 0.0001), and semi-closed their eyes (Pr—p < 0.001 and Ab—p < 0.0001) for longer compared to baseline. Four hours post-recovery (Analgesia) the rabbits remained lying down for longer (Pr and Ab—p < 0.0001), compared to the baseline and 24h post. Putting weight on and raising limbs was more frequent at 24h post (Pr—p < 0.0001 and Ab—p < 0.01) compared to baseline (Table 3).

The rabbits remained less in the quadrupedal position (p < 0.001), ate less carrots (p < 0.0001), interacted less with the pinecone (p < 0.0001), and winced more (p < 0.001) only in the absence of the observer at one hour post-recovery (Pain) (Table 3).

In the presence of the observer, 4h post-recovery (Analgesia), compared to Baseline and 24h post, the rabbits remained more frequently at the back of the cage (p = 0.0058) and less frequently at the front (p = 0.0058) and remained seated for a shorter time (p < 0.0001). The duration of body self-cleaning was shorter at the 1h post-recovery (Pain) than at 24h post (p < 0.001). When the observer was absent the rabbits remained less frequently in the normal posture after 4h post-recovery (Analgesia) compared to Baseline (p < 0.05) (Table 3).

### Differences in pain behaviours between the presence and absence of the observer

In the presence of the familiar female observer, compared to the absence of the observer, there was a higher frequency and/or duration of the following behaviours: inactive at all time-points (Baseline—p < 0.0001, Pain—p < 0.01, Analgesia—p = 0.0195; 24h post—p < 0.05) and lie down posture (p < 0.01), react (p < 0.05) and wince (p < 0.05) at 24h post recovery. Otherwise, in the presence of the observer, at all time-points the rabbits remained active (Baseline—p < 0.0001, Pain—p < 0.01, Analgesia—p < 0.05; 24h post—p < 0.05), in normal posture (24h post) (p < 0.05) and seated (p < 0.05) and lying down (Analgesia) (p < 0.001) for a shorter period compared to the absence of the observer (Table 3). When the observer was present, there was a lower frequency of explore (Baseline) (p < 0.01), flinch (Pain) (p < 0.05), put weight on and raise the affected limb (Analgesia) (p < 0.05), shake body (p < 0.05) and suspended limb at 24h (p < 0.05). When the observer was present there was a lower duration of explore (Baseline) (p < 0.01), suspended limb (Pain) (p < 0.05), lick the affected area (Analgesia) (p < 0.05), and normal posture at 24h post (p < 0.05) (Table 3).

### Behaviours that did not change over time or in the presence of the observer

Lying on one side and bipedal postures and the behaviours rotating jump, shake the head, dig, drink, eat feed, gnaw, press limbs, stretch, scratch ear, ingest cecotropes, punch, head self-cleaning, twitch, stagger, fall, tremble, try to get up, writhe, eyes closed and eyes not visible, ears completely lowered, semi-lowered, erect, and ‘scissor’ ears did not alter over time or between the presence and absence of the observer (S2 Table).

## Discussion

This study corroborated previously reported postoperative pain behaviours in rabbits after ovariohysterectomy (OVH) [9] and demonstrated the presence of different and apparently specific orthopaedic pain behaviours not identified in previous studies on soft tissue surgery [9, 12, 15]. Another finding that confirms the study hypothesis was that the presence of the observer influenced both the preoperative behaviour of the pain-free state and the postoperative pain behavioural expression.

The lack of support from the affected limb in the presence of musculoskeletal injuries is a common finding in rabbits and other species [31]. The movement of put weight and raise up the limb was identified in the current study as a specific behaviour after orthopaedic surgery in rabbits. The lack of a number of pain behaviours that have been previously observed after soft tissue surgery, such as arching of the back and pressing the abdomen towards the floor [9] most likely indicated that these behaviours are probably specific to abdominal pain since they were not identified in this study.

Press limbs, suspend the limb, ‘scissor’ ears, scratch ears, punch, ingest cecotropes, tremble, and try to get up behaviours were identified in this study, but were not described in the previous OVH ethogram [9], however, there were no differences in their frequency of occurrence or duration over time in this study. Some of the behaviours observed, such as suspend the limb, try to get up, stagger, and close the eyes occurred exclusively following surgery (S2 Table), suggesting that their expression is related to postoperative pain, as reported for stagger and closed eyes [9].

In the immediate post-recovery period, both before and after the rescue analgesia, the rabbits were less active, and thus rarely hopped and changed position in the cage and changed posture. This is similar to that observed after soft tissue surgeries without the presence of an observer [9, 13]. Decreased activity after surgery minimizes pain and discomfort and potentially accelerates recovery [12], has a protective function [9], and is an important indicator of pain in this species [15, 27]. However, in rabbits evaluated in a previous study in the presence of an observer [8], there was no difference in activity scores between the immediate and late postoperative periods, contrary to the present study, where the activity was lower even with the observer present. This is likely to be because in the previous study the rabbits were under the effect of analgesics, as well as due to differences in acclimation period, housing, type of surgery, and anaesthetic protocol.

Following rescue analgesia, the rabbits remained in the back of the cage for longer when the observer was present, which may reflect a natural escape behaviour from a potential threat [32], since in both the current study and previous research [9, 12], when the observer was absent, there were no differences in cage positioning.

In previous studies, weight loss in the days following surgery has been related to pain [9, 27]. Although weight loss is a delayed evaluation [19], as it is directly related to food consumption, it can be evaluated in real time through appetite. However, if food is available *ad libitum*, the measurement of consumption over a short period of time may not be representative of the animals’ pain state. This limitation was observed in the current study, since the behaviour of eating the food did not change over time which demonstrates that evaluation of appetite may not differentiate pain-free vs painful states. Alternatively, the interest in a piece of carrot as a motivational item reduced during post-recovery in the absence of the observer. As carrot is more palatable, fresh, and less frequently offered than feed, it seems to stimulate the appetite and allows evaluation of appetite in real time. The same approach has been used in cats by offering more palatable and different food to what they were conventionally given [20].

In addition to eat the carrot, interact with the pinecone and explore appear to be important for defining pain-free rabbits, because they decreased postoperatively. Likewise, pain following soft tissue surgery in rabbits reduced exploratory behaviour [9, 13]. The presence of the observer reduced explore behaviour at baseline, showing the inhibitory influence of the observer even in pain-free animals.

The introduction of a pinecone stimulated play behaviour and interaction with a new object, which resembles the interaction with toys [9, 13, 15]. The inclusion of new objects stimulates the innate activity of animals, even in environments with restricted space, such as cages [33]. However, it is important to replace objects frequently to maintain interaction and positive effect, otherwise the interaction is reduced once the rabbit is familiar with the object [34]. In this study a new pinecone was offered at each recording period in the absence of the observer, to arouse the curiosity of the animals. The interactions with motivational items in the current study were good indicators of pain-free state and well-being, as playful behaviour represents potential positive emotions [35]. In contrast, the reduction in these interactions indicates painful states after surgery.

Although the interaction with motivational items occurred at low frequency and duration even at baseline, this behaviour is apparently sensitive and specific to pain, occurring in most rabbits at baseline, but in the minority of rabbits after surgery.

Suspend limb and lick the affected area were the most expected behaviours after limb surgery, however they were apparently masked by the presence of the observer. Wince behaviour is important to identify both orthopaedic, as in the current study, and soft tissue postoperative pain [9, 12].

Another indicator that forms part of the behavioural repertoire of painful rabbit seems to be semi-closed eyes, previously described in the Rabbit Grimace Scale [10], as well as other laboratory animal species [36, 37], cats [38, 39], horses [40], and sheep [41]. Similarly, as expected, the presence of open eyes decreased in the presence of pain in this study.

Ear position was important indicator of pain in rabbits when assessed only from images [10, 42] using a 3-point scale. However, like in previous studies -[9, 12], the current study showed no pain-related changes in ear position when assessed by duration, even with the introduction of a catheter into the auricular right ear vein. Ear position is an apparently non-specific pain behaviour because it may be influenced by environmental stimuli and other motives, such as during self-cleaning behaviour.

As rabbits are sociable animals in nature and interact with each other, caged rabbits exhibit more frequent body self-cleaning due to lack of environmental stimulation [43]. In contrast to observations following other types of surgery [9, 12, 13], this behaviour did not reduce after orthopaedic surgery in this study, and the possible explanations may be because orthopaedic surgery performed in the limb does not prevent rabbits from grooming nor reduce the motivation to groom as seen with visceral pain [9, 13] and/or because grooming may be a displacement activity post-surgery that distracts rabbits from pain.

Licking of the affected area did not change over time, in contrast to previous studies in rabbits [10] and other species [20, 23] experiencing pain. Factors other than pain may influence this behaviour and the higher than expected occurrence at baseline could be due to the shaving performed the day before, as described in rats submitted to abdominal shaving [24] and rabbits with a jugular catheter [13].

React and shake the body behaviours were not altered in pain situations in the current and other studies [9, 12], but they were affected by the presence of the observer. Reactive behaviour is related to the animal’s reactivity to stimuli, which increased when the observer was present at 24 hours post-recovery. Otherwise the observer inhibited shaking, a behaviour related to comfort [43].

In addition to their presence, the observer’s sex may influence the behavioural expression of the animals. The expression of pain through facial expressions and the threshold of nociceptive tests were masked by the presence of a male observer, but not female observers in rodents [28]. This is apparently related to the presence of androstenone and androstadienone in axillary secretions found predominantly in males. The current study excluded this factor as the observer was female; however, different results could be found if the observer were male.

Another factor that may have attenuated the differences between the presence and absence of the observer was that the rabbits were familiar with the observer, who was responsible for the handling and feeding of the animals during the acclimation period. Rats demonstrate a preference for familiar researchers [26, 44] and even superficial handling interaction between laboratory rats and researchers reduced rat anxiety scores [26].

Positive interactions between humans and other animals in experimental conditions reduce stress and facilitate identification of abnormal behaviours related to pain and disease [45]. In the current study, it is challenging to assess whether the interaction was positive or negative. Familiarity through feeding and animal care procedures prior to surgery can have a positive effect [26], however, in this study the interaction observed at 24 hours post-recovery, was aggressive, suggesting this interaction was apparently negative. After rabbits have been submitted to stressful situations such as surgery and administration of analgesics, the effect of the presence of the observer was more evident in a higher number of behaviours (active, inactive, react, shake body, wince and suspend limb behaviours and normal and lying down postures) at 24 hours postoperatively compared to the other moments.

As described in other species, appetite would be expected to increase after rescue analgesia, both due to reduced pain [21, 46, 47] and through the effect of opioids [48]. This was not the case in the current study, and was likely to be due to morphine and meloxicam were insufficient to relieve pain after surgery or because morphine caused sedation.

The time-point after rescue analgesia was defined as three hours after administration to avoid the effect of sedation observed post-morphine injection, since in a pilot study intense sedation of the animals was observed one hour after morphine. Therefore, 3h after morphine administration it would be expected that the analgesic effect would be present, but with little effect of sedation [49]. Sedation was not assessed because there are no validated tools to quantify sedation in rabbits as reported for dogs [50] and observation of this parameter was beyond the scope of this study. A possible effect of sedation influencing the expression of postoperative pain behaviour cannot be disregarded in rabbits; cats undergoing ketamine administration have been falsely diagnosed with pain [51]. Little is known about the effects of morphine on pain behaviours in leporine species. At doses of 3 to 5 mg/kg, morphine sedated rabbits [52]; the lower dose resulted in analgesia for 240 minutes and reduced activity for up to 300 minutes [49], when the postoperative rescue analgesia time-point was assessed in the current study.

Although buprenorphine is the most commonly used opioid in rabbits [16] and in behavioural studies [8, 13, 15], this drug is not commercially available in Brazil. Thus, the choice of analgesic protocol was based on the fact that the 1 mg/kg dose of meloxicam was the most effective dosage in rabbits submitted to OVH [9]. Since the orthopaedic surgery induces severe pain, morphine 2 mg/kg was included in the analgesic protocol, as this is an effective opioid analgesic [5, 46]. The analgesic protocol used in this study was similar to that described in other species to validate pain scales [20, 21].

Pre- and trans-operative analgesia was based on pethidine and fentanyl respectively. These opioids apparently guarantee sufficient trans-operative analgesia because there were no pain-related autonomic trans-operative changes. As pethidine and fentanyl are short acting opioids, their effects were abated shortly after surgery; this facilitated expression and identification of postoperative pain behaviours in this study. Morphine and meloxicam apparently prevented hyperalgesia up to 24h after anaesthetic recovery, because by this time behaviours were similar to baseline. Carprofen and indomethacin prevented experimentally induced hyperalgesia in cats and rabbits respectively [53, 54] and morphine reversed hyperalgesia in rabbits [54].

The maintenance of postoperative analgesia with meloxicam for a few days would be more appropriate than tramadol only. However, this was an opportunistic study and because one of the aims of the other study was to measure acute-phase protein, the anti-inflammatory effect produced by long-term treatment with meloxicam would interfere in this analysis. Dogs submitted to orthopaedic surgery exhibited the most intense degree of pain in the initial postoperative hours [18]. Considering that at 24h, the rabbits´ behaviours were similar to baseline, it appears the rabbits were not suffering sufficient pain to require analgesia, other than tramadol, at 24h post-recovery.

In a previous study [9], rabbits were less active in the morning compared to the afternoon. In another study rabbit behaviours did not change over the course of the day [13]. In the current study, the animals underwent surgery from early morning until early afternoon, they acted as their own controls and time-point intervals were the same. In this study, this probably compensated for the effect of circadian rhythm on behavioural variables.

A 20-minute period has been suggested as representative for evaluating behaviour in rabbits [9], however this is too long and impractical for clinical evaluation. Brevity is an important quality of pain assessment tools [22]. Behaviour assessment of the pain-free state is essential to compare against the painful state and, therefore, identify pain-related behaviours. However, if there is no stimulus in the pain-free state, the animal may rest most of the time and this low activity may be falsely identified as pain. Alternatively, if environmental enrichment is provided to stimulate activity, the animal could become accustomed and lose interest, as reported in the previous study in rabbits submitted to OVH, where even when assessing the rabbit´s behaviour for 20 minutes, the interaction with the toy was sporadic [9]. Although the evaluation time was shorter in the current study, it seems that the inclusion of new motivational items in every behaviour assessment optimized the analysis of the playing behaviour and allowed expression of pain behaviours.

### Limitations

One of the limitations of the current study was the different female-male ratio and the non-evaluation of the female estrous cycle. This was an opportunistic study, which followed the recommendations for developing pain evaluation methods [7], by using animals that had already undergone surgical stimulation, instead of promoting suffering in other animals for this exclusive purpose. Previous studies showed that due to their hormonal cycling, female rabbits are more likely to be antisocial compared to male rabbits [55]. Female mice showed a higher mechanical threshold than males [56], and bitches tend to protect the surgical wound area more than male dogs [46].

A control group of rabbits submitted only to anaesthesia could identify the exclusive anaesthetic effects on post-intervention behaviour [19]. Previous studies also did not include control groups, however, most of the behaviours identified in the current study were similar to those described previously in rabbits [9, 13, 15] and in other species suffering pain [18]. A future study should be performed to address possible anaesthesia-related behavioural changes.

Videos should ideally be assessed by a blind evaluator to avoid expectation bias [57], however this risk was minimized because among the methodologies used in behavioural studies, the recording of ethogram based on frequency and duration of behaviours is one of the least affected by expectation bias [58]. The order of filming in the absence and presence of the observer was not randomized. This decision was taken as it was impossible to know how much time was necessary for the rabbit to return to the original behaviour after the observer had left. Similarly, the five-minute period before filming began may not have been sufficient to adapt the rabbit to the absence of the observer.

Although in the absence of the observer there was no eye contact with the animal, as the observer was in another room, one cannot rule out the possibility that the rabbit could smell the observer. To minimize this limitation, the observer did not use any perfume during the filming period.

## Conclusion

Orthopaedic surgery pain in rabbits reduces activity, changes in different postures and position around the cage, and behaviours of hopping and explore, as well as increasing the presence of semi-closed eyes in both the presence and absence of the observer. In the absence of the observer at the moment of likely greatest pain post-recovery (i.e. 1h), the rabbits remained for less time in the quadrupedal posture, ate less carrot, interacted less with the pinecone, and winced more frequently. The behaviours of suspend the affected limb and put weight on and raise the affected limb appear to be specific to orthopaedic pain. The presence of the female and familiar observer before surgery reduced activity and exploratory behaviour, leading to the false impression of pain in a pain-free state. After surgery, the frequency of wince increased and flinch, lick the affected area, suspend the limb, and put weight on and raise the affected limb decreased in the presence of the observer, which suggests that the rabbits were masking signs of pain, which is likely to affect sensitivity of these behaviours for the diagnosis of pain, possibly leading to false negative results.

## Supporting information

S1 Table Video examples of each perioperative behaviour in rabbits submitted to orthopaedic surgery. (DOCX)

S2 Table Median and interquartile range (Q1 –Q3) of behaviours of the 28 rabbits undergoing orthopaedic surgery that did not alter over time or between the presence and absence of observer. Frequency (F) in number of occurrences and duration (D) in seconds in each of the 300 second (five minute) observations of behaviours that were not different over time or between the absence and presence of the observer, observed in the 28 rabbits undergoing orthopaedic surgery in the moments before surgery (Baseline); 1 hour after recovery from anaesthesia (Pain); 4 hours after recovery and 3 hours after rescue analgesia (Analgesia); and 24 hours after recovery from anaesthesia (24h post).
(DOCX)

S1 Data Data of the ethogram. (XLSX)

S1 Fig (TIF)

S1 Video (MP4)

S2 Video (MP4)

S3 Video (MP4)

S4 Video (MP4)

S5 Video (MP4)

S6 Video (MP4)

S7 Video (MP4)

S8 Video (MP4)

S9 Video (MP4)

S10 Video (MP4)

S11 Video (MP4)

S12 Video (MP4)

S13 Video (MP4)

S14 Video (MP4)

S15 Video (MP4)

S16 Video (MP4)

S17 Video (MP4)

S18 Video (MP4)

S19 Video (MP4)

S20 Video (MP4)

S21 Video (MP4)

S22 Video (MP4)

S23 Video (MP4)

S24 Video (MP4)

S25 Video (MP4)

S26 Video (MP4)

S27 Video (MP4)

S28 Video (MP4)

S29 Video (MP4)

S30 Video (MP4)

S31 Video (MP4)

S32 Video (MP4)

S33 Video (MP4)

S34 Video (MP4)

S35 Video (MP4)

S36 Video (MP4)

S37 Video (MP4)

S38 Video (MP4)

S39 Video (MP4)

S40 Video (MP4)

S41 Video (MP4)

S42 Video (MP4)

S43 Video (MP4)

S44 Video (MP4)

S45 Video (MP4)

S46 Video (MP4)

S47 Video (MP4)

S48 Video (MP4)

S49 Video (MP4)

S50 Video (MP4)

## References

[1] Williams ACDC, Craig KD. Updating the definition of pain. Pain. 2016;157: 2420–2423. 10.1097/j.pain.0000000000000613 27200490

[2] Great Britain, Home Office, Great Britain, Parliament, House of Commons. Annual statistics of scientific procedures on living animals: Great Britain 2018. 2019. https://assets.publishing.service.gov.uk/government/uploads/system/uploads/attachment_data/file/835935/annual-statistics-scientific-procedures-living-animals-2018.pdf

[3] Benato L, Rooney NJ, Murrell JC. Pain and analgesia in pet rabbits within the veterinary environment: a review. Vet Anaesth Analg. 2019;46: 151–162. 10.1016/j.vaa.2018.10.007 30737017

[4] Uhlig C, Krause H, Koch T, De Abreu MG, Spieth PM. Anesthesia and monitoring in small laboratory mammals used in anesthesiology, respiratory and critical care research: A systematic review on the current reporting in top-10 impact factor ranked journals. PLoS One. 2015;10: 1–22. 10.1371/journal.pone.0134205 26305700 PMC4549323

[5] Flecknell P. Analgesics in Small Mammals. Vet Clin North Am—Exot Anim Pract. 2018;21: 83–103. 10.1016/j.cvex.2017.08.003 29146033

[6] Izer JM, LaFleur RA, Weiss WJ, Wilson RP. Development of a Pain Scoring System for Use in Sheep Surgically Implanted with Ventricular Assist Devices. J Investig Surg. 2018;1939: 1–10. 10.1080/08941939.2018.1457191 29641275

[7] Hawkins P. Recognizing and assessing pain, suffering and distress in laboratory animals: A survey of current practice in the UK with recommendations. Lab Anim. 2002;36: 378–395. 10.1258/002367702320389044 12396281

[8] Divincenti L, Meirelles LAD, Westcott RA. Safety and clinical effectiveness of a compounded sustained-release formulation of buprenorphine for postoperative analgesia in New Zealand white rabbits. J Am Vet Med Assoc. 2016;248: 795–801. 10.2460/javma.248.7.795 27003021

[9] Leach MC, Allweiler S, Richardson C, Roughan J V., Narbe R, Flecknell PA. Behavioural effects of ovariohysterectomy and oral administration of meloxicam in laboratory housed rabbits. Res Vet Sci. 2009;87: 336–347. 10.1016/j.rvsc.2009.02.001 19303122

[10] Keating SCJ, Thomas AA, Flecknell PA, Leach MC. Evaluation of EMLA Cream for Preventing Pain during Tattooing of Rabbits: Changes in Physiological, Behavioural and Facial Expression Responses. PLoS One. 2012;7: 1–11. 10.1371/journal.pone.0044437 22970216 PMC3436883

[11] Banchi P, Quaranta G, Ricci A, Von Degerfeld MM. Reliability and construct validity of a composite pain scale for rabbit (CANCRS) in a clinical environment. PLoS One. 2020;15: 1–12. 10.1371/journal.pone.0221377 32352960 PMC7192371

[12] Farnworth MJ, Walker JK, Schweizer KA, Chuang CL, Guild SJ, Barrett CJ, et al Potential behavioural indicators of post-operative pain in male laboratory rabbits following abdominal surgery. Anim Welf. 2011;20: 225–237.

[13] Schnellbacher RW, Divers SJ, Comolli JR, Beaufrère H, Maglaras CH, Andrade N, et al Effects of intravenous administration of lidocaine and buprenorphine on gastrointestinal tract motility and signs of pain in New Zealand white rabbits after ovariohysterectomy. Am J Vet Res. 2017;78: 1359–1371. 10.2460/ajvr.78.12.1359 29182394

[14] Lehner PN. Design and execution of animal behavior research: an overview. Journal of animal science. 1987 pp. 1213–1219. 10.2527/jas1987.6551213x 3320003

[15] Weaver LA, Blaze CA, Linder DE, Andrutis KA, Karas AZ. A model for clinical evaluation of perioperative analgesia in rabbits (Oryctolagus cuniculus). J Am Assoc Lab Anim Sci. 2010;49: 845–851. 21205451 PMC2994053

[16] Coulter CA, Flecknell PA, Leach MC, Richardson CA. Reported analgesic administration to rabbits undergoing experimental surgical procedures. BMC Vet Res. 2011;7 10.1186/1746-6148-7-12 21338514 PMC3058034

[17] Benato L, Murrell JC, Blackwell EJ, Saunders R, Rooney N. Analgesia in pet rabbits: A survey study on how pain is assessed and ameliorated by veterinary surgeons. Vet Rec. 2020;186: 603 10.1136/vr.105071 32303663

[18] Rialland P, Authier S, Guillot M, del Castillo JRE, Veilleux-Lemieux D, Frank D, et al Validation of Orthopedic Postoperative Pain Assessment Methods for Dogs: A Prospective, Blinded, Randomized, Placebo-Controlled Study. PLoS One. 2012;7: 1–10. 10.1371/journal.pone.0049480 23166681 PMC3500314

[19] Dunbar ML, David EM, Aline MR, Lofgren JL. Validation of a behavioral ethogram for assessing postoperative pain in Guinea pigs (cavia porcellus). J Am Assoc Lab Anim Sci. 2016;55: 29–34. 26817977 PMC4747008

[20] Brondani JT, Mama KR, Luna SPL, Wright BD, Niyom S, Ambrosio J, et al Validation of the English version of the UNESP-Botucatu multidimensional composite pain scale for assessing postoperative pain in cats. BMC Vet Res. 2013;9 10.1186/1746-6148-9-143 23867090 PMC3722032

[21] de Oliveira FA, Luna SPL, do Amaral JB, Rodrigues KA, Sant’Anna AC, Daolio M, et al Validation of the UNESP-Botucatu unidimensional composite pain scale for assessing postoperative pain in cattle. BMC Vet Res. 2014;10: 1–14. 10.1186/1746-6148-10-1 25192598 PMC4172785

[22] Reid J, Nolan AM, Hughes JML, Lascelles D, Pawson P, Scott EM. Development of the short-form Glasgow Composite Measure Pain Scale (CMPS-SF) and derivation of an analgesic intervention score. Anim Welf. 2007;16: 97–104.

[23] Holton L, Pawson P, Nolan A, Reid J, Scott EM. Development of a behaviour-based scale to measure acute pain in dogs. Vet Rec. 2001;148: 525–531. 10.1136/vr.148.17.525 11354645

[24] Roughan J V., Flecknell PA. Behavioural effects of laparotomy and analgesic effects of ketoprofen and carprofen in rats. Pain. 2001;90: 65–74. 10.1016/s0304-3959(00)00387-0 11166971

[25] Weary DM, Niel L, Flower FC, Fraser D. Identifying and preventing pain in animals. Appl Anim Behav Sci. 2006;100: 64–76. 10.1016/j.applanim.2006.04.013

[26] Van Driel KS, Talling JC. Familiarity increases consistency in animal tests. Behav Brain Res. 2005;159: 243–245. 10.1016/j.bbr.2004.11.005 15817187

[27] Cooper CS, Metcalf-Pate KA, Barat CE, Cook JA, Scorpio DG. Comparison of side effects between buprenorphine and meloxicam used postoperatively in Dutch belted rabbits (Oryctolagus cuniculus). J Am Assoc Lab Anim Sci. 2009;48: 279–285. 19476717 PMC2696831

[28] Sorge RE, Martin LJ, Isbester KA, Sotocinal SG, Rosen S, Tuttle AH, et al Olfactory exposure to males, including men, causes stress and related analgesia in rodents. Nat Methods. 2014;11: 629–632. 10.1038/nmeth.2935 24776635

[29] Lester LS, Fanselow MS. Exposure to a cat produces opioid analgesia in rats. Behav Neurosci. 1985;99: 756–759. 10.1037//0735-7044.99.4.756 3843739

[30] Hubrecht Carter. The 3Rs and Humane Experimental Technique: Implementing Change. Animals. 2019;9: 754 10.3390/ani9100754 31575048 PMC6826930

[31] Gallego M, Villaluenga JE. Coxofemoral luxation in pet rabbits: nine cases. J Small Anim Pract. 2019;60: 631–635. 10.1111/jsap.12866 29920673

[32] McBride EA. Small prey species’ behaviour and welfare: implications for veterinary professionals. J Small Anim Pract. 2017;58: 423–436. 10.1111/jsap.12681 28513850

[33] Poggiagliolmi S, Crowell-Davis SL, Alworth LC, Harvey SB. Environmental enrichment of New Zealand White rabbits living in laboratory cages. J Vet Behav Clin Appl Res. 2011;6: 343–350. 10.1016/j.jveb.2010.12.001

[34] Johnson CA, Pallozzi WA, Geiger L, Szumiloski JL, Castiglia L, Dahl NP, et al The effect of an environmental enrichment device on individually caged rabbits in a safety assessment facility. Contemp Top Lab Anim Sci. 2003;42: 27–30. 14510521

[35] Boissy A, Manteuffel G, Jensen MB, Moe RO, Spruijt B, Keeling LJ, et al Assessment of positive emotions in animals to improve their welfare. Physiol Behav. 2007;92: 375–397. 10.1016/j.physbeh.2007.02.003 17428510

[36] Langford DJ, Bailey AL, Chanda ML, Clarke SE, Drummond TE, Echols S, et al Coding of facial expressions of pain in the laboratory mouse. Nat Methods. 2010;7: 447–449. 10.1038/nmeth.1455 20453868

[37] Sotocinal SG, Sorge RE, Zaloum A, Tuttle AH, Martin LJ, Wieskopf JS, et al The Rat Grimace Scale: A partially automated method for quantifying pain in the laboratory rat via facial expressions. Mol Pain. 2011 10.1186/1744-8069-7-55 21801409 PMC3163602

[38] Finka LR, Luna SP, Brondani JT, Tzimiropoulos Y, McDonagh J, Farnworth MJ, et al Geometric morphometrics for the study of facial expressions in non-human animals, using the domestic cat as an exemplar. Sci Rep. 2019 10.1038/s41598-019-46330-5 31285531 PMC6614427

[39] Evangelista MC, Watanabe R, Leung VSY, Monteiro BP, O’Toole E, Pang DSJ, et al Facial expressions of pain in cats: the development and validation of a Feline Grimace Scale. Sci Rep. 2019;9: 19128 10.1038/s41598-019-55693-8 31836868 PMC6911058

[40] Dalla Costa E, Minero M, Lebelt D, Stucke D, Canali E, Leach MC. Development of the Horse Grimace Scale (HGS) as a Pain Assessment Tool in Horses Undergoing Routine Castration. Hillman E, editor. PLoS One. 2014;9: e92281 10.1371/journal.pone.0092281 24647606 PMC3960217

[41] McLennan KM, Rebelo CJB, Corke MJ, Holmes MA, Leach MC, Constantino-Casas F. Development of a facial expression scale using footrot and mastitis as models of pain in sheep. Appl Anim Behav Sci. 2016;176: 19–26. 10.1016/j.applanim.2016.01.007

[42] Hedenqvist P, Trbakovic A, Thor A, Ley C, Ekman S, Jensen-Waern M. Carprofen neither reduces postoperative facial expression scores in rabbits treated with buprenorphine nor alters long term bone formation after maxillary sinus grafting. Res Vet Sci. 2016;107: 123–131. 10.1016/j.rvsc.2016.05.010 27473985

[43] Podberscek AL, Blackshaw JK, Beattie AW. The behaviour of group penned and individually caged laboratory rabbits. Appl Anim Behav Sci. 1991;28: 353–363. 10.1016/0168-1591(91)90167-V

[44] Davis H, Taylor AA, Norris C. Preference for familiar humans by rats. Psychon Bull Rev. 1997;4: 118–120. 10.3758/BF03210783

[45] Zulkifli I. Review of human-animal interactions and their impact on animal productivity and welfare. J Anim Sci Biotechnol. 2013;4: 1–7. 10.1186/2049-1891-4-1 23855920 PMC3720231

[46] Wagner AE, Worland GA, Glawe JC, Hellyer PW. Multicenter, randomized controlled trial of pain-related behaviors following routine neutering in dogs. J Am Vet Med Assoc. 2008;233: 109–115. 10.2460/javma.233.1.109 18593318

[47] Brondani JT, Luna SPL, Minto BW, Santos BPR, Beier SL, Matsubara LM, et al Validade e responsividade de uma escala multidimensional para avaliação de dor pós-operatória em gatos. Arq Bras Med Vet e Zootec. 2012;64: 1529–1538. 10.1590/S0102-09352012000600019

[48] Dodman NH, Zuroff S. Reverse tolerance to the stimulant effects of morphine in horses. J Equine Vet Sci. 1984;4: 233–236. 10.1016/S0737-0806(84)80153-7

[49] Barter LS, Kwiatkowski A. Thermal threshold testing for evaluation of analgesics in New Zealand white rabbits. J Am Assoc Lab Anim Sci. 2013;52: 44–47. 23562032 PMC3548200

[50] Wagner MC, Hecker KG, Pang DSJ. Sedation levels in dogs: A validation study. BMC Vet Res. 2017;13: 1–8. 10.1186/s12917-016-0931-1 28420386 PMC5395740

[51] Buisman M, Wagner MC, Hasiuk MMM, Prebble M, Law L, Pang DSJ. Effects of ketamine and alfaxalone on application of a feline pain assessment scale. J Feline Med Surg. 2016;18: 643–651. 10.1177/1098612X15591590 26088567 PMC10816383

[52] Flecknell PA. The relief of pain in laboratory animals. Lab Anim. 1984;18: 147–160. 10.1258/002367784780891226 6146743

[53] Taylor PM, Steagall PVM, Dixon MJ, Ferreira TH, Luna SPL. Carprofen and buprenorphine prevent hyperalgesia in a model of inflammatory pain in cats. Res Vet Sci. 2007;83: 369–375. 10.1016/j.rvsc.2007.01.007 17363018

[54] Dong H, Sun H, Magal E, Ding X, Kumar GN, Chen JJ, et al Inflammatory pain in the rabbit: A new, efficient method for measuring mechanical hyperalgesia in the hind paw. J Neurosci Methods. 2008;168: 76–87. 10.1016/j.jneumeth.2007.09.028 18022246

[55] Mullan SM, Main DCJ. Behaviour and personality of pet rabbits and their interactions with their owners. Vet Rec. 2007;160: 516–520. 10.1136/vr.160.15.516 17435098

[56] Vacca V, Marinelli S, Pieroni L, Urbani A, Luvisetto S, Pavone F. Higher pain perception and lack of recovery from neuropathic pain in females: A behavioural, immunohistochemical, and proteomic investigation on sex-related differences in mice. Pain. 2014;155: 388–402. 10.1016/j.pain.2013.10.027 24231652

[57] Tuyttens FAM, de Graaf S, Heerkens JLT, Jacobs L, Nalon E, Ott S, et al Observer bias in animal behaviour research: Can we believe what we score, if we score what we believe? Anim Behav. 2014;90: 273–280. 10.1016/j.anbehav.2014.02.007

[58] Tuyttens FAM, Stadig L, Heerkens JLT, Van laer E, Buijs S, Ampe B. Opinion of applied ethologists on expectation bias, blinding observers and other debiasing techniques. Appl Anim Behav Sci. 2016 10.1016/j.applanim.2016.04.019

