# Supplementary material for: Postoperative pain behaviours in rabbits following orthopaedic surgery and effect of observer presence
Source: PLoS One. 2020 Oct 22;15(10):e0240605. doi: 10.1371/journal.pone.0240605 (PMC7580914; doi:10.1371/journal.pone.0240605)
Supplement: S1 Table — (DOCX) [file pone.0240605.s001.docx]

| **Behaviour** | **Description** | **Link to the video** |
| --- | --- | --- |
| Active | Moves and/or moves around | S1 Video |
| Inactive | Remains stationary | S2 Video |
| Front | Front of the cage | S3 Video |
| Back | Back of the cage | S4 Video |
| Change position | Moves around the cage: front to back or back to front | S5 Video |
| Normal | Flexes pelvic limbs under the hip, thoracic limbs under the body; abdomen and chest not supported on cage floor | S6 Video |
| Seated | Sits with vertically extended thoracic limbs | S7 Video |
| Lying down | Lies with the abdomen and chest in contact with the cage floor and horizontally extended thoracic limbs | S8 Video |
| Lying on one side | Lies on one side of the body with horizontally extended thoracic limbs | S9 Video |
| Quadrupedal | In the quadrupedal position with the four limbs extended vertically; abdomen not in contact with the cage floor | S10 Video |
| Bipedal | Supported on both pelvic limbs; thoracic limbs not touching the floor | S11 Video |
| Change posture | Alternates between different postures | S12 Video |
| Completely lowered | Parallel to the vertebral column | S13 Video |
| Semi-lowered | Semi-lowered (positioned between fully lowered and erect) | S14 Video |
| Erect | Erect, perpendicular to the spine | S15 Video |
| ‘Scissor’ ears | Each ear moves to different positions | S16 Video |
| Open | Fully open | S17 Video |
| Semi-closed | Semi-closed | S18 Video |
| Closed | Closed | S19 Video |
| Not visible | Not possible to visualize the eyes | S20 Video |
| Hop | Hops to move around with both pelvic limbs at the same time | S21 Video |
| Rotating jump | Jumps performing a 180º or 360º rotation with both pelvic limbs at the same time | S22 Video |
| React | Moves head and/or body sharply in response to environmental stimulus (e.g., sound stimulus) | S23 Video |
| Shake body | Shakes the whole body | S24 Video |
| Shake the head | Shakes only the head | S25Video |
| Dig | Digs the floor | S26 Video |
| Explore | Sniffs the cage floor and/or walls and/or bars curiously | S27 Video |
| Gnaw | Gnaws the floor or bars of the cage | S28 Video |
| Press limbs | Presses limbs strongly against the cage floor | S29 Video |
| Stretch | Stretches the body | S30 Video |
| Scratch ear | Scratches the ears with the limbs | S31 Video |
| Punch | Extends the thoracic limbs horizontally, quickly alternating between right and left | S32 Video |
| Interact with pinecone | Interacts with the pinecone (chews, gnaws, pushes with the thoracic limbs) | S33 Video |
| Eat carrot | Eats the carrot | S34 Video |
| Drink | Drinks water from the water trough | S35 Video |
| Eat feed | Eats feed from the feeder | S36 Video |
| Ingest cecotropes | Ingests anal cecotropes | S37 Video |
| Head | Head and ears | S38 Video |
| Body | Body, including abdomen and limbs (except affected limb) | S39 Video |
| Lick affected area | Licks affected region | S40Video |
| Twitch* | Presents spasms in the skin of the back | S41 Video |
| Flinch* | Moves body quickly dorsally and for no apparent reason | S42 Video |
| Wince* | Shrinks back and closes eyes | S43 Video |
| Stagger* | Partially loses balance | S44 Video |
| Fall* | Totally loses balance; falls to the floor | S45 Video |
| Tremble | Presents tremors observed in head and ears | S46 Video |
| Suspend limb | Keeps the affected limb suspended | S47 Video |
| Put weight on and raise the affected limb | Raises and puts weight on the affected limb repeatedly | S48 Video |
| Try to get up | Tries to get up, but remains in a normal posture or lying down | S49 Video |
| Writhe* | Contracts the muscles of the abdomen | S50 Video |
| * indicates pain behaviours taken from [9] | |  |
