# Supplementary material for: Postoperative pain behaviours in rabbits following orthopaedic surgery and effect of observer presence
Source: PLoS One. 2020 Oct 22;15(10):e0240605. doi: 10.1371/journal.pone.0240605 (PMC7580914; doi:10.1371/journal.pone.0240605)
Supplement: S2 Table — Frequency (F) in number of occurrences and duration (D) in seconds in each of the 300 second (five minute) observations of behaviours that were not different over time or between the absence and presence of the observer, observed in the 28 rabbits undergoing orthopaedic surgery in the moments before surgery (Baseline); 1 hour after recovery from anaesthesia (Pain); 4 hours after recovery and 3 hours after rescue analgesia (Analgesia); and 24 hours after recovery from anaesthesia (24h post). (DOCX) [file pone.0240605.s002.docx]

| **Behaviour** | **F/D** | **Observer** | **Baseline** | **Pain** | **Analgesia** | **24h post** |
| --- | --- | --- | --- | --- | --- | --- |
| *Posture* | | | | | | |
| Lying on side | D | Pr | 0 (0-0) | 0 (0-0) | 0 (0-0) | 0 (0-0) |
|  |  | Ab | 0 (0-0) | 0 (0-0) | 0 (0-0) | 0 (0-0) |
| Bipedal | F | Pr | 0 (0-1) | 0 (0-0) | 0 (0-0) | 0 (0-1) |
|  |  | Ab | 0 (0-2) | 0 (0-0) | 0 (0-0) | 0 (0-1) |
|  | D | Pr | 0 (0-2) | 0 (0-0) | 0 (0-0) | 0 (0-1) |
|  |  | Ab | 0 (0-3) | 0 (0-0) | 0 (0-0) | 0 (0-1) |
| *Position of the ears* | | | | | | |
| Completely lowered | D | Pr | 0 (0-0) | 0 (0-0) | 0 (0-0) | 0 (0-0) |
|  |  | Ab | 0 (0-0) | 0 (0-0) | 0 (0-0) | 0 (0-0) |
| Semi-lowered | D | Pr | 0 (0-115) | 45 (0-300) | 20 (0-300) | 0 (0-115) |
|  |  | Ab | 0 (0-52) | 125 (0-298) | 16 (0-300) | 0 (0-174) |
| Erect | D | Pr | 300 (115-300) | 134 (0-300) | 66 (0-300) | 300 (40-300) |
|  |  | Ab | 300 (147-300) | 83 (0-300) | 90 (0-300) | 293 (48-300) |
| ‘Scissor’ ears | D | Pr | 0 (0-0) | 0 (0-0) | 0 (0-0) | 0 (0-0) |
|  |  | Ab | 0 (0-0) | 0 (0-0) | 0 (0-0) | 0 (0-0) |
| *Eye opening* | | | | | | |
| Closed | D | Pr | 0 (0-0) | 0 (0-0) | 0 (0-0) | 0 (0-0) |
|  |  | Ab | 0 (0-0) | 0 (0-0) | 0 (0-32) | 0 (0-0) |
| Not visible | D | Pr | 3 (0-57) | 7 (0-129) | 0 (0-52) | 17 (0-74) |
|  |  | Ab | 14 (0-97) | 6 (0-104) | 0 (0-103) | 5 (0-52) |
| *Typical rabbit behaviours* | | | | | | |
| Rotating jump | F | Pr | 0 (0-0) | 0 (0-0) | 0 (0-0) | 0 (0-0) |
|  |  | Ab | 0 (0-0) | 0 (0-0) | 0 (0-0) | 0 (0-0) |
| Shake head | F | Pr | 0 (0-1) | 0 (0-0) | 0 (0-0) | 0 (0-0) |
|  |  | Ab | 0 (0-1) | 0 (0-0) | 0 (0-0) | 0 (0-0) |
| Dig | F | Pr | 0 (0-0) | 0 (0-0) | 0 (0-0) | 0 (0-0) |
|  |  | Ab | 0 (0-0) | 0 (0-0) | 0 (0-0) | 0 (0-0) |
|  | D | Pr | 0 (0-0) | 0 (0-0) | 0 (0-0) | 0 (0-0) |
|  |  | Ab | 0 (0-0) | 0 (0-0) | 0 (0-0) | 0 (0-0) |
| Gnaw | F | Pr | 0 (0-0) | 0 (0-0) | 0 (0-0) | 0 (0-0) |
|  |  | Ab | 0 (0-1) | 0 (0-0) | 0 (0-0) | 0 (0-0) |
|  | D | Pr | 0 (0-0) | 0 (0-0) | 0 (0-0) | 0 (0-0) |
|  |  | Ab | 0 (0-1) | 0 (0-0) | 0 (0-0) | 0 (0-0) |
| Press limbs | F | Pr | 0 (0-0) | 0 (0-0) | 0 (0-0) | 0 (0-0) |
|  |  | Ab | 0 (0-0) | 0 (0-0) | 0 (0-0) | 0 (0-0) |
| Stretch | F | Pr | 0 (0-0) | 0 (0-0) | 0 (0-0) | 0 (0-0) |
|  |  | Ab | 0 (0-0) | 0 (0-0) | 0 (0-0) | 0 (0-0) |
| Scratch ear | F | Pr | 0 (0-0) | 0 (0-0) | 0 (0-0) | 0 (0-1) |
|  |  | Ab | 0 (0-0) | 0 (0-0) | 0 (0-0) | 0 (0-3) |
|  | D | Pr | 0 (0-0) | 0 (0-0) | 0 (0-0) | 0 (0-2) |
|  |  | Ab | 0 (0-0) | 0 (0-0) | 0 (0-0) | 0 (0-3) |
| Punch | F | Pr | 0 (0-1) | 0 (0-0) | 0 (0-0) | 0 (0-1) |
|  |  | Ab | 0 (0-1) | 0 (0-1) | 0 (0-0) | 0 (0-1) |
| *Physiological behaviours* | | | | | | |
| Drink | F | Pr | 0 (0-0) | 0 (0-0) | 0 (0-0) | 0 (0-0) |
|  |  | Ab | 0 (0-0) | 0 (0-0) | 0 (0-0) | 0 (0-0) |
|  | D | Pr | 0 (0-0) | 0 (0-0) | 0 (0-0) | 0 (0-0) |
|  |  | Ab | 0 (0-0) | 0 (0-0) | 0 (0-0) | 0 (0-0) |
| Eat feed | F | Pr | 0 (0-0) | 0 (0-0) | 0 (0-0) | 0 (0-0) |
|  |  | Ab | 0 (0-0) | 0 (0-0) | 0 (0-0) | 0 (0-0) |
|  | D | Pr | 0 (0-0) | 0 (0-0) | 0 (0-0) | 0 (0-0) |
|  |  | Ab | 0 (0-0) | 0 (0-0) | 0 (0-0) | 0 (0-0) |
| Ingest cecotropes | F | Pr | 0 (0-0) | 0 (0-0) | 0 (0-0) | 0 (0-0) |
|  |  | Ab | 0 (0-0) | 0 (0-0) | 0 (0-0) | 0 (0-0) |
|  | D | Pr | 0 (0-0) | 0 (0-0) | 0 (0-0) | 0 (0-0) |
|  |  | Ab | 0 (0-0) | 0 (0-0) | 0 (0-0) | 0 (0-0) |
| *Self-cleaning* | | | | | | |
| Head | F | Pr | 0 (0-0) | 0 (0-0) | 0 (0-0) | 0 (0-0) |
|  |  | Ab | 0 (0-0) | 0 (0-0) | 0 (0-0) | 0 (0-1) |
|  | D | Pr | 0 (0-0) | 0 (0-0) | 0 (0-0) | 0 (0-0) |
|  |  | Ab | 0 (0-0) | 0 (0-0) | 0 (0-0) | 0 (0-1) |
| *Pain-related behaviors* | | | | | | |
| Twitch | F | Pr | 0 (0-0) | 0 (0-0) | 0 (0-0) | 0 (0-0) |
|  |  | Ab | 0 (0-0) | 0 (0-0) | 0 (0-0) | 0 (0-0) |
| Stagger | F | Pr | 0 (0-0) | 0 (0-0) | 0 (0-0) | 0 (0-0) |
|  |  | Ab | 0 (0-0) | 0 (0-0) | 0 (0-0) | 0 (0-0) |
| Fall | F | Pr | 0 (0-0) | 0 (0-0) | 0 (0-0) | 0 (0-0) |
|  |  | Ab | 0 (0-0) | 0 (0-0) | 0 (0-0) | 0 (0-0) |
| Tremble | F | Pr | 0 (0-0) | 0 (0-1) | 0 (0-0) | 0 (0-0) |
|  |  | Ab | 0 (0-0) | 0 (0-1) | 0 (0-0) | 0 (0-0) |
|  | D | Pr | 0 (0-0) | 0 (0-34) | 0 (0-0) | 0 (0-0) |
|  |  | Ab | 0 (0-0) | 0 (0-64) | 0 (0-0) | 0 (0-0) |
| Try to get up | F | Pr | 0 (0-0) | 0 (0-1) | 0 (0-0) | 0 (0-0) |
|  |  | Ab | 0 (0-0) | 0 (0-1) | 0 (0-0) | 0 (0-0) |
| Writhe | F | Pr | 0 (0-0) | 0 (0-0) | 0 (0-0) | 0 (0-0) |
|  |  | Ab | 0 (0-0) | 0 (0-0) | 0 (0-0) | 0 (0-0) |
|  | | | | | | |
|  |  |  |  |  |  |  |
