# Supplementary figures and images for: Postoperative pain behaviours in rabbits following orthopaedic surgery and effect of observer presence
Source: PLoS One. 2020 Oct 22;15(10):e0240605. doi: 10.1371/journal.pone.0240605 (PMC7580914; doi:10.1371/journal.pone.0240605)

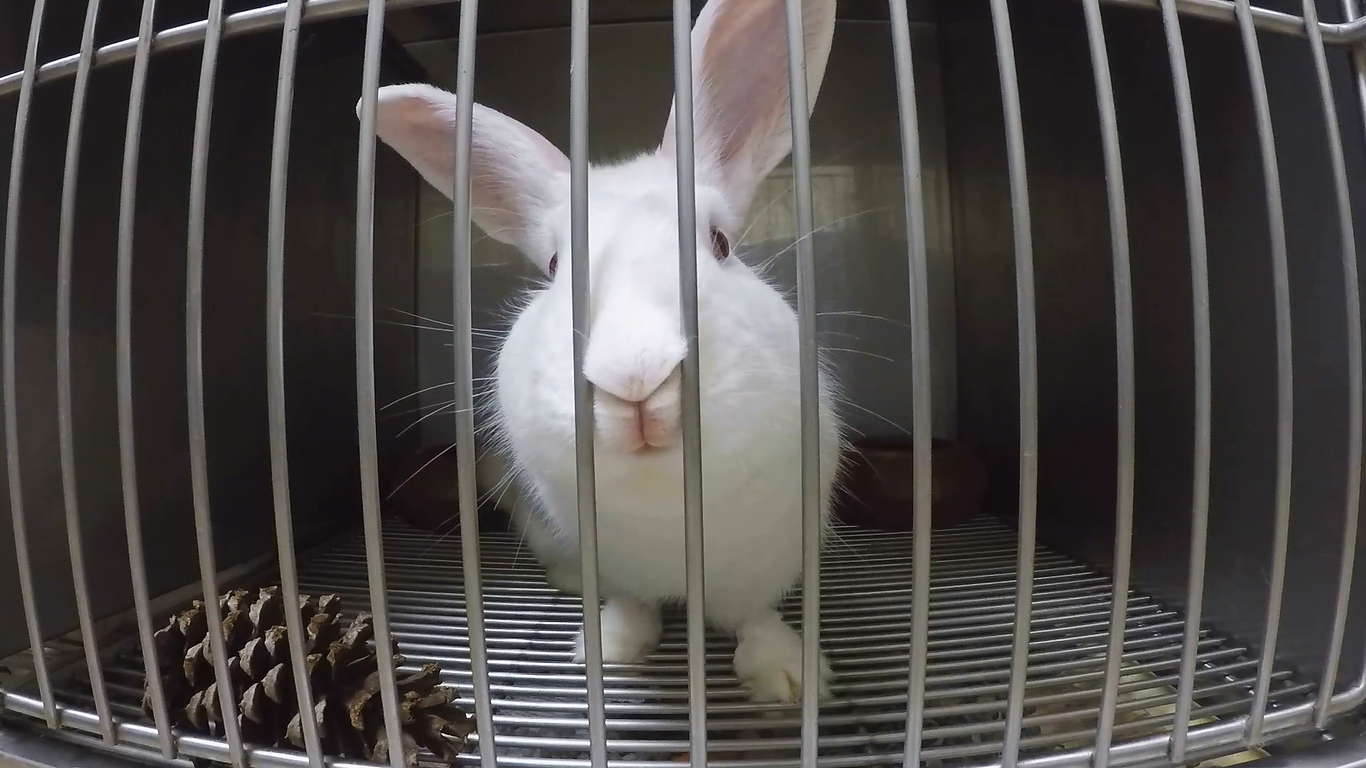

Supplement: S1 Fig — (TIF) [file pone.0240605.s004.tif]
